# Phytochemical-Loaded Thermo-responsive Liposome for Synergistic Treatment of Methicillin-Resistant *Staphylococcus aureus* Infection

**DOI:** 10.34133/bmr.0159

**Published:** 2025-03-13

**Authors:** Sidi Zheng, Xinshu Zou, Yanru Wei, Xilong Cui, Shuang Cai, Xiubo Li, Zhiyun Zhang, Yanhua Li

**Affiliations:** ^1^ College of Veterinary Medicine, Northeast Agricultural University, Harbin 150030, PR China.; ^2^ Heilongjiang Key Laboratory for Animal Disease Control and Pharmaceutical Development, Harbin 150030, PR China.; ^3^ Feed Research Institute, Chinese Academy of Agricultural Sciences, Beijing 100081, PR China.

## Abstract

The ever-increasing emergence and prevalence of multidrug-resistant bacteria accelerate the desire for the development of new antibacterial strategies. Although antibacterial phytochemicals are a promising approach for long-term treatment of resistant bacteria, their low antibacterial activity and poor solubility hinder their practical applications. Here, the natural antibacterial compound sanguinarine (SG) together with gallic acid–ferrous coordination nanoparticles (GA-Fe(II) NPs) was encapsulated in a near-infrared (NIR)-activated thermo-responsive liposome. By virtue of the photothermal effect of GA-Fe(II) NPs, the nanoplatform released SG on demand upon NIR irradiation. Additionally, the heat can boost the Fenton reaction triggered by GA-Fe(II) NPs to generate hydroxyl radicals and perform sterilization. By coupling with photothermal therapy, chemodynamic therapy, and SG-based pharmacotherapy, the platform showed enhanced antibacterial efficiency and an antibiofilm effect toward methicillin-resistant *Staphylococcus aureus* and reduced the risk of developing new bacterial resistance. This antibacterial system displayed excellent antibacterial activity in a methicillin-resistant *S. aureus*-caused skin abscess, demonstrating its potential clinical application. Moreover, transcription analysis clarified that the platform achieved a synergistic antibacterial effect by attacking the cell membrane, inducing energy metabolism disorder, inhibiting nucleic acid synthesis, etc. The developed NIR-controlled phytochemical-loaded platform offers new possibilities for killing antibiotic-resistant bacteria and avoiding bacterial resistance, making it contributory in the fields of anti-infective therapy and precision medicine.

## Introduction

*Staphylococcus aureus* is a major pathogen that threatens public health. As a versatile pathogen, *S. aureus* can induce a variety of diseases ranging from skin and soft tissue infection to life-threatening sepsis, leading to high morbidity and mortality clinically [1,2]. Over the past decades, antibiotics have been a cornerstone of the modern medical system for bacterial infection treatment. However, the misuse of antibiotics inevitably boosted the rise and dissemination of multidrug-resistant (MDR) bacteria, like methicillin-resistant *S. aureus* (MRSA), which is impotent to most conventional antibiotics and contributes to an increase in annual mortality [3,4]. Discouragingly, introducing new antibiotics such as daptomycin to combat drug resistance seems in vain since bacteria can develop resistance to newly introduced antibiotics expeditiously [5–7]. Given the severity of the issue, alternative therapeutic strategies with a low possibility of developing bacterial resistance are in urgent demand, especially antibiotic-free ones.

Natural plant products represent a viable option in response to the current antibacterial dilemma [8,9]. Currently, the potential of natural antimicrobial compounds for drug-resistant strains has been intensively investigated, such as alkaloids [10]. Compared with conventional antibiotics, alkaloids exhibit bactericidal activities through a complex mechanism, which reduces the chance of resistance development [11–14]. Additionally, these natural alkaloids are readily accessible and abundant, offering a substantial resource for developing new antibacterial agents. Although promising, most natural antimicrobial compounds present a lower antibacterial activity than conventional antibiotics. To achieve a satisfactory bactericidal effect, a high dose of natural compounds is needed. Moreover, the poor solubility of most natural alkaloids causes low bioavailability and further decreases their bactericidal effect, which hinders their practical applications in the replacement of antibiotics [15,16].

Nanotechnology-based controllable drug release systems provide versatile platforms for revolutionizing the antibacterial application of bactericides. They can realize controllable delivery of antibacterial agents in the infectious site by different triggers, including endogenous biochemical stimuli (pH, enzymes, and H_2_O_2_) and exogenous physical stimuli (heat and light), and in turn improve the bioavailability and antibacterial efficacy of drugs [17,18]. Among them, near-infrared (NIR)-light-mediated nanosystems possess a higher tissue penetration depth and precise remote control ability [19–21]. Therefore, they have been proposed as a promising candidate in controlling the on-demand delivery of antibacterial agents. Meanwhile, photothermal therapy (PTT) activated by NIR can induce physical damage of pathogenic cells such as membrane disruption, protein denaturation, and eventually bacterial death without generating bacterial resistance [22–24]. Recently, the development of an NIR-responsive nanodrug delivery system enabled the combination of PTT and pharmacotherapy in MRSA infection [25,26]. Nevertheless, efficient PPT often necessitates either high laser power densities or prolonged laser exposure to sustain a high temperature in bacterial regions, potentially harming adjacent healthy tissues and diminishing the compliance of PTT [27,28].

Recently, iron–polyphenol coordination polymer nanoparticles have gained popularity in PTT applications owing to their stability, biocompatibility, and structural diversity [29]. Moreover, ferrous ions (Fe^2+^) can initiate the Fenton reaction, continuously converting H_2_O_2_ into highly cytotoxic hydroxyl radicals (•OH), leading to cell death [30]. At bacterial infection sites, the chemotaxis of proinflammatory immune cells causes elevated reactive oxygen species (ROS) levels, facilitating the elimination of bacterial infections by converting endogenous H_2_O_2_ into •OH in the presence of iron, a process known as chemodynamic therapy (CDT) [31,32]. Importantly, the heat produced by PTT enhances ROS generation in CDT, thereby improving antibacterial efficacy [33]. Considering that, integration of a ferrous–polyphenol complex with natural compounds can potentially realize regaining the antibacterial effect of natural compounds at low concentrations while also enhancing antibacterial effects through synergistic PTT/CDT/pharmacotherapy and slowing the development of bacterial resistance.

**Fig. 1.**
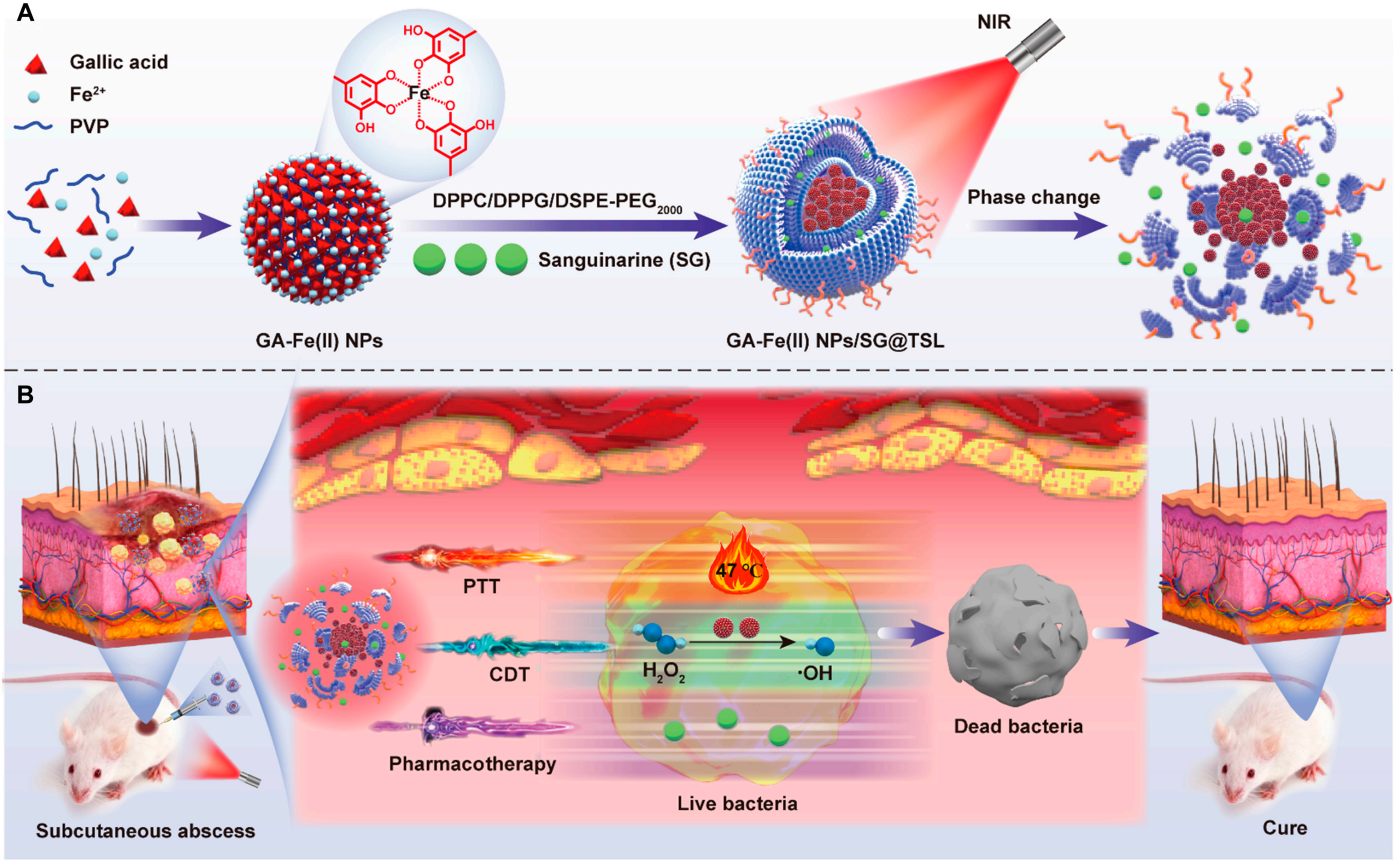
Illustration of (A) GA-Fe(II) NPs/SG@TSL fabrication and (B) its synergistic photothermal, chemodynamic, and pharmacological therapy against methicillin-resistant *Staphylococcus aureus* (MRSA) infection upon near-infrared (NIR) activation. GA-Fe(II) NPs, gallic acid–ferrous coordination nanoparticles; SG, sanguinarine; TSL, temperature-sensitive liposome; PVP, polyvinylpyrrolidone; DPPC, 1,2-dipalmitoyl-*sn*-glycero-3-phosphocholine; DPPG, 1,2-dipalmitoyl-*sn*-glycero-3-phospho-*rac*-(1-glycerol) sodium salt; DSPE-PEG_2000_, 1,2-distearoyl-*sn*-glycero-3-phosphoethanolamine-*N*-[methoxy(polyethylene glycol)-2000]; PTT, photothermal therapy; CDT, chemodynamic therapy.

Sanguinarine (SG), a bioactive alkaloid extracted from *Macleaya cordata*, exhibits broad-spectrum bactericidal action against both gram-positive and gram-negative bacteria, even MDR strains [34]. Therefore, in this study, an NIR-activated temperature-sensitive-liposome (TSL)-based nanomedicine system that is capable of co-delivery of SG and ferrous–polyphenol complexes was designed to achieve synergistic PTT/CDT/pharmacotherapy against MRSA (Fig. 1). In this system, ultrasmall gallic acid–ferrous nanoparticles (GA-Fe(II) NPs) were constructed via a simple mix of Fe^2+^ with gallic acid (GA). The obtained nanoparticles possessed continuable catalytic activity in converting H_2_O_2_ to •OH. Upon NIR irradiation, the high heat generated by the GA-Fe(II) NPs could promote the phase transition of the bilayers of TSLs, leading to the rapid release of SG. Meanwhile, the photothermal effect of GA-Fe(II) NPs could enhance drug permeation into bacteria by destroying membrane permeability and bolster CDT efficacy by accelerating the generation of ROS. Taken together, this phytochemical-loaded platform enables NIR-triggered drug release, supporting photothermal, chemodynamic, and pharmacological synergistic therapy, thereby presenting a safe and effective antibiotic-free strategy for MDR infections.

## Materials and Methods

### Materials

GA and doxorubicin (DOX) were sourced from Macklin Biochemical Co., Ltd (Shanghai, China). Polyvinylpyrrolidone, 1,2-dipalmitoyl-*sn*-glycero-3-phosphocholine (DPPC), and coumarin 6 (CM 6) were sourced from Aladdin Biochemical Technology Co., Ltd (Shanghai, China). Iron dichloride tetrahydrate (FeCl_2_·4H_2_O) and 1,2-distearoyl-*sn*-glycero-3-phosphoethanolamine-*N*-[methoxy(polyethylene glycol)-2000] (DSPE-PEG_2000_) were obtained from Yuan Ye Biochemical Co., Ltd (Shanghai, China). 1,2-Dipalmitoyl-*sn*-glycero-3-phospho-*rac*-(1-glycerol) sodium salt (DPPG) was obtained from AVT Pharmaceutical Technology Co., Ltd (Shanghai, China). SG was purchased from Chengdu Herbpurify Co., Ltd (Chengdu, China). Chloroform and methanol were obtained from Kermel Analytical Reagent Co., Ltd (Tianjin, China). Trypticase soy broth (TSB) was provided by Hope Bio-Technology Co., Ltd (Qingdao, China).

### Bacterial culture

An MRSA ATCC 43300 colony on a trypticase soy agar (TSA) plate was cultured overnight in TSB medium at 37 °C. Subsequently, the bacterial suspensions were diluted 1:1,000 and cultured until they reached the log phase for subsequent use. The bacterial concentration was assessed by monitoring the optical density at 600 nm (OD 600) via an ultraviolet spectrophotometer.

### Minimal inhibitory concentration assay

The minimal inhibitory concentration (MIC) of SG was determined using the standard microdilution method. Briefly, MRSA were cultured to the logarithmic phase and then diluted to a ratio of 1:1,000. Subsequently, a 2-fold serial dilution of SG was conducted in a total volume of 200 μl. The plates were incubated at 37 °C for 24 h, and the MIC was defined as the minimum concentration of the agent that fully inhibited visible bacterial growth.

### Resistance development studies

The MRSA culture (200 μl, 1 × 10^5^ colony-forming units [CFU] ml^−1^) was joined to a 96-well plate containing different concentrations of SG. Daptomycin and vancomycin served as positive control. The bacterial MIC was recorded following 24-h incubation at 37 °C. Subsequently, 10 μl of MRSA culture from the sublethal MIC plate was diluted in 10 ml of TSB, and the procedure was repeated. The cultures underwent serial passaging for a duration of 30 d.

### Synthesis of GA-Fe(II) NPs

GA-Fe(II) NPs were synthesized using the method described by Dong et al. [30]. In short, FeCl_2_·4H_2_O (23 mg) and polyvinylpyrrolidone (80 mg) were dispersed with degassed deionized (DI) water. After that, 1 ml of GA (10 mg ml^−1^) was gradually introduced into the above solution and stirred under a nitrogen atmosphere for 24 h. The resulting GA-Fe(II) NPs were then purified using a dialysis membrane. The as-synthesized GA-Fe(II) NPs were freeze-dried for further analysis and usage.

### Characterization of GA-Fe(II) NPs

The ultraviolet absorption spectrum of GA-Fe(II) NPs was measured by an ultraviolet spectrophotometer (EVOLUTION201, China), and their chemical structure was analyzed via a Fourier transform infrared spectrometer (Thermo Scientific Nicolet iS20, USA). The particle sizes of GA-Fe(II) NPs were tested by a Malvern Particle Sizer (Malvern Panalytical, USA). The morphologies of GA-Fe(II) NPs were examined using transmission electron microscopy (TEM; H-7650, Hitachi, Japan). The element valence of Fe in GA-Fe(II) NPs was analyzed using x-ray photoelectron spectroscopy (Thermo ESCALAB 250XI, USA), while thermogravimetric analysis quantified the Fe-loading capacity.

### Photothermal performance of GA-Fe(II) NPs

Aqueous solutions of GA-Fe(II) NPs at varying concentrations (0.2, 0.4, 0.8, 1.2, or 2.4 mg ml^−1^) were subjected to 808-nm NIR laser irradiation at a power density of 1.5 W cm^−2^ for 10 min. An infrared imaging device monitored the solutions’ temperatures every 10 s.

### Photothermal stability of GA-Fe(II) NPs

The photothermal stability of GA-Fe(II) NPs was assessed using NIR laser irradiation at 1.5 W cm^−2^ over 5 on–off cycles. Briefly, the GA-Fe(II) NPs (0.4 mg ml^−1^) were subjected to 808-nm NIR laser irradiation for 10 min to reach a stable temperature, and then the laser was turned off to allow them to cool naturally. The irradiation cycle was conducted 5 times. An infrared imaging device recorded the temperature during both heating and cooling phases. To obtain the accurate ultraviolet absorption of GA-Fe(II) NPs when the cycles just stopped, the GA-Fe(II) NP solution was immediately scanned with an ultraviolet spectrophotometer at a full wavelength.

### Hydroxyl radical (•OH) generation evaluation

•OH production was evaluated according to the method described previously. Briefly, a mixed solution containing GA-Fe(II) NPs (0.4 mg ml^−1^), methylene blue (MB, 15 μg ml^−1^), and H_2_O_2_ (1 mM) was cultured at 37 °C for a defined time. At each preset time, the absorbance values of the solution at 665 nm were determined. The levels of •OH production under NIR laser irradiation was studied by exposing the mixed solution to an 808-nm laser for 15 min while maintaining the temperature at 47 °C.

### Stability study of GA-Fe(II) NPs

GA-Fe(II) NP aqueous solutions were placed at a stable temperature environment of 4 and 25 °C, respectively. At predefined time intervals, the dynamic diameters of GA-Fe(II) NPs were measured using a Malvern Particle Sizer.

### Preparation of GA-Fe(II) NPs/SG@TSL

The TSLs were prepared using the thin lipid film hydration technique. DPPC, DPPG, and DSPE-PEG_2000_ in a weight ratio of 36:9:10 were dissolved in a chloroform-and-methanol mixture (2:1, *v*/*v*) along with SG (0.25 mg ml^−1^). The mixture was continuously stirred at 45 °C for 1 h and evaporated to form a homogeneous lipid film. The obtained lipid film was subsequently hydrated with GA-Fe(II) NP solution for 5 h. The as-prepared TSLs were sonicated, centrifuged, and then purified through a 0.45-μm polycarbonate membrane and Sephadex G-100 columns. Ultraviolet spectrophotometry was carried out to quantify the content of SG in GA-Fe(II) NPs/SG@TSL.

### Characterization of GA-Fe(II) NPs/SG@TSL

For the characterization of GA-Fe(II) NPs/SG@TSL, the size distribution was measured by a Malvern Particle Sizer, and the morphologies were observed via TEM. The photothermal effect of GA-Fe(II) NPs/SG@TSL was studied by monitoring the temperature changes of the GA-Fe(II) NPs/SG@TSL solution (0.4 mg ml^−1^ GA-Fe(II) NPs) upon irradiation as described above. Meanwhile, the •OH generation activities of GA-Fe(II) NPs/SG@TSL (0.4 mg ml^−1^ GA-Fe(II) NPs) was investigated as described above.

### Drug release behavior

The release of SG from GA-Fe(II) NPs/SG@TSL was determined by dialyzing GA-Fe(II) NPs/SG@TSL against buffers (pH = 5.5) containing Tween 80 (1%, *w*/*w*) at 37 °C with gentle agitation. Following a 1-h incubation, GA-Fe(II) NPs/SG@TSL was exposed to 10-min irradiation using an 808-nm NIR laser at 1.5 W cm^−2^. At designed time intervals, the amount of SG in the released medium was determined by an ultraviolet spectrophotometer. The solution without NIR irradiation served as the control.

### Physiological stability study of GA-Fe(II) NPs/SG@TSL

GA-Fe(II) NPs/SG@TSL was incubated in phosphate-buffered saline (pH = 7.4) at 37 °C to simulate physiological conditions. At predefined time intervals, the dynamic diameter and morphological changes of GA-Fe(II) NPs/SG@TSL were determined using a Malvern Particle Sizer and TEM, respectively.

### Cytotoxicity assessment of TSLs

The cytotoxic effects of TSLs were evaluated utilizing the Cell Counting Kit-8 (CCK-8) assay. Murine fibroblast cells (L929 cells) were cultured in a 96-well plate for 24 h at 37 °C. Then, cells were incubated for 24 h in a fresh medium with varying concentrations of TSLs. Then the medium was replaced with CCK-8 solution in each well. After incubation, the absorbance at 450 nm of all samples was determined using a microplate reader.

### Hemolytic assay of TSLs

Red blood cells (RBCs) of sheep were isolated through centrifugation, washed, and resuspended with saline. Then, various concentrations of TSLs were introduced to the RBC stock dispersion and co-cultured at 37 °C for 3 h. For comparison, saline and DI water were appointed as the negative and positive controls, respectively. The supernatant’s absorbance at 576 nm was measured using a microplate reader following centrifugation.

### Bacterial uptake study

CM 6 served as the model fluorescent drug to study the bacterial uptake of TSLs. Firstly, CM 6 and GA-Fe(II) NPs coloaded TSLs were prepared (GA-Fe(II) NPs/CM 6@TSL) using a procedure similar to that for GA-Fe(II) NPs/SG@TSL. MRSA was cultured with GA-Fe(II) NPs/CM 6@TSL at 37 °C for 3 h. Subsequently, the culture medium was centrifuged, and the MRSA culture was washed and resuspended in saline. The obtained samples were analyzed using flow cytometry.

Moreover, the time-dependent MRSA internalization was further investigated. MRSA were incubated with GA-Fe(II) NPs/CM 6@TSL (0.2 μg ml^−1^ CM 6) for 3, 6, and 12 h and then were detected by flow cytometry.

### Antibacterial properties of GA-Fe(II) NPs/SG@TSL in vitro

The antibacterial efficiency of GA-Fe(II) NPs/SG@TSL was assessed via plate counting. Briefly, MRSA suspensions in log phase were diluted to 10^6^ CFU ml^−1^ with saline. The diluted MRSA were incubated at 37 °C for 12 h with shaking under various treatment conditions: (a) control, (b) SG (12 μg ml^−1^), (c) TSLs, (d) SG@TSL, (e) GA-Fe(II) NPs@TSL, (f) GA-Fe(II) NPs/SG@TSL, (g) GA-Fe(II) NPs/SG@TSL + H_2_O_2_, (h) NIR only, (i) SG (12 μg ml^−1^) + NIR, (j) TSLs + NIR, (k) SG@TSL + NIR, (l) GA-Fe(II) NPs@TSL + NIR, (m) GA-Fe(II) NPs/SG@TSL + NIR, and (n) GA-Fe(II) NPs/SG@TSL + H_2_O_2_ + NIR. The treated MRSA were diluted and spread onto a TSA plate. The colonies were counted after a 24-h incubation period. Here, the treatments involving NIR were exposed to irradiation via a 808-nm NIR laser at 1.5 W cm^−2^ for 10 min. The concentration of H_2_O_2_ used in this study was 100 μM.

### Live/dead staining assay

The MRSA viability in test samples was qualitatively evaluated using a LIVE/DEAD BacLight Bacterial Viability Kit. MRSA were isolated by centrifugation and washed with saline after different treatments. Then, MRSA were sequentially stained with propidium iodide (PI) for 5 min and SYTO 9 for 15 min. Afterward, their fluorescence images were obtained via an inverted fluorescence microscope.

### Morphological characterization of MRSA

The morphology of MRSA following various treatments was examined using scanning electron microscopy (SEM). Briefly, MRSA samples underwent centrifugation at 10,000 rpm for 5 min following various treatments and were subsequently washed 3 times with saline. After that, the MRSA were fixed with 2.5% glutaraldehyde and dehydrated. The final samples were dried and sprayed with gold before being tested by SEM.

### Ultrastructural effects of MRSA

MRSA were exposed to saline, H_2_O_2_, SG, GA-Fe(II) NPs/SG@TSL + NIR, or GA-Fe(II) NPs/SG@TSL + H_2_O_2_ + NIR for 12 h at 37 °C. Afterward, MRSA were harvested by centrifugation at 10,000 rpm. The obtained MRSA samples were sequentially fixed with 2.5% glutaraldehyde and then postfixed with 1% osmium tetroxide. The samples were stained using 2% uranyl acetate. Micrographs of MRSA were examined by TEM.

### Penetration of TSLs into biofilm

Fluorescent drug DOX-loaded liposomes were prepared to trace the distribution in biofilm. The MRSA suspension (2 ml, 1 × 10^5^ CFU ml^−1^) was placed in petri dishes and cultured for 48 h at 37 °C. The established biofilms were cultured with GA-Fe(II) NPs/DOX@TSL (equivalent DOX of 10 μg ml^−1^) for 3 h without shaking. Subsequently, the samples were gently washed with saline and stained with SYTO 9. The distribution of GA-Fe(II) NPs/DOX@TSL inside the biofilm was visualized via confocal laser scanning microscopy (CLSM).

### Characterization of biofilm morphology

The mature biofilms were exposed to SG (24 μg ml^−1^), H_2_O_2_ (100 μM), GA-Fe(II) NPs/SG@TSL, GA-Fe(II) NPs/SG@TSL + NIR, and GA-Fe(II) NPs/SG@TSL + H_2_O_2_ + NIR, respectively. Meanwhile, the established biofilm mixed with TSB media only was defined as the control group. In each NIR irradiation group, the biofilms were exposed to an 808-nm NIR laser at 1.5 W cm^−2^ for 10 min following 3-h incubation. After a 12-h incubation period, all samples were washed and stained with SYTO 9/PI for 15 min. CLSM was employed to visualize the live and dead MRSA in the residual biofilms.

### Crystal violet assay

The biofilms were subjected to various treatments for 12 h, consistent with the conditions used in the characterization of biofilm morphology. Then, the biofilms were adequately fixed with methanol and stained with 0.1% crystal violet for 30 min. The stained biofilms were rinsed with saline for 3 times. After that, the biofilms were dispersed in 33% acetic acid, and their absorbance values were detected by a microplate reader at 595 nm.

### Murine skin infection model

Female BALB/c mice (20 to 22 g) were sourced from the Second Affiliated Hospital of Harbin Medical University. A subcutaneous abscess was established in mice by subcutaneous injection of 100 μl of MRSA suspension (1 × 10^8^ CFU ml^−1^) into the right side of the back. The experimental animal research ethics committee of Northeast Agricultural University (SRM-11) approved the animal experiment protocols, ensuring compliance with relevant ethical regulations. After 24 h, the mice with subcutaneous abscesses were randomly assigned to 10 groups and treated with various formulations (100 μl) by subcutaneous injection into the infected abscess, including (a) saline, (b) SG (24 μg ml^−1^), (c) SG@TSL, (d) GA-Fe(II) NPs@TSL, (e) GA-Fe(II) NPs/SG@TSL, (f) saline + NIR, (g) SG (24 μg ml^−1^) + NIR, (h) SG@TSL + NIR, (i) GA-Fe(II) NPs@TSL + NIR, and (j) GA-Fe(II) NPs/SG@TSL + NIR. Three hours postinjection, the abscess areas were exposed to an 808-nm NIR laser at 1 W cm^−2^ for 10 min in each NIR irradiation group. The abscesses were dynamically photographed in the whole treatment period. After 8 d of treatment, the mice were sacrificed by euthanasia. The infected mouse skins were excised, fixed with 4% formaldehyde, and stained with hematoxylin and eosin (H&E) for histopathological analysis. To calculate the MRSA load in the infection region, the suspension of skin tissue was plated on a TSA plate. Bacterial colonies were counted following 24-h incubation at 37 °C. Furthermore, the fixed skins underwent immunohistochemical staining for tumor necrosis factor alpha (TNF-α) and interleukin-6 (IL-6) to assess inflammation levels at the infection site. In addition, vancomycin was employed as a positive drug for comparison of the therapeutic effects of the developed GA-Fe(II) NPs/SG@TSL; 100 μl (1 mg ml^−1^) of vancomycin was subcutaneously injected into the mice with subcutaneous abscesses. The level of abscess recovery and MRSA load in the infection region were used to evaluate the treatment efficacy.

### Biosafety assessment in vivo

To assess the biosafety of GA-Fe(II) NPs/SG@TSL, BALB/c mice were assigned to 3 groups and administered with saline, SG, and GA-Fe(II) NPs/SG@TSL + NIR. The administration procedure and treatment period of each group followed the treatment process in vivo. After 8 d of progress, the mice were sacrificed, and blood samples were collected from each group. The blood parameters and blood biochemical index were measured. Afterward, the major organs from mice were dissected and subjected to H&E staining for histopathological examination.

### Transcriptome analysis

Gene expression of MRSA was assessed upon exposure to SG (12 μg ml^−1^) or GA-Fe(II) NPs/SG@TSL + H_2_O_2_ + NIR. Meanwhile, MRSA without treatment served as the control group. The treatment process of GA-Fe(II) NPs/SG@TSL + H_2_O_2_ + NIR was followed with that in the in vitro antibacterial study. Total RNA was independently isolated and purified from 3 samples per condition for RNA sequencing. Shanghai Personal Biotechnology Co. Ltd conducted the Illumina sequencing using the NovaSeq 6000 platform. Postsequencing, the raw data quality was assessed, and Cutadapt (v1.15) was employed to filter the raw data. Quality-filtered reads were aligned to the reference assembly accession (GCF_-_003052445.1) via a Bowtie 2 (v 2.2.6). Gene read counts were calculated using HTSeq (v 0.9.1), and DESeq was employed to identify differentially expressed genes (DEGs) across samples, defined by |log_2_(fold change)| > 1 and *P* < 0.05. Thereafter, DEGs were performed on an enrichment analysis of Gene Ontology terms. Meanwhile, the clusterProfiler (3.4.4) software was employed to perform the enrichment analysis in the Kyoto Encyclopedia of Genes and Genomes (KEGG) of DEGs, emphasizing pathways with marked enrichment (*P* < 0.05).

### Statistical analysis

The data in this study are presented as mean ± standard deviation. GraphPad Prism 8 (GraphPad Software, Inc., La Jolla, CA, USA) was utilized for statistical analyses. The *t* test and one-way analysis of variance were performed to assess the statistical differences. *P* < 0.05 was defined to be significant.

## Results and Discussion

### Characterization of GA-Fe(II) NPs

**Fig. 2.**
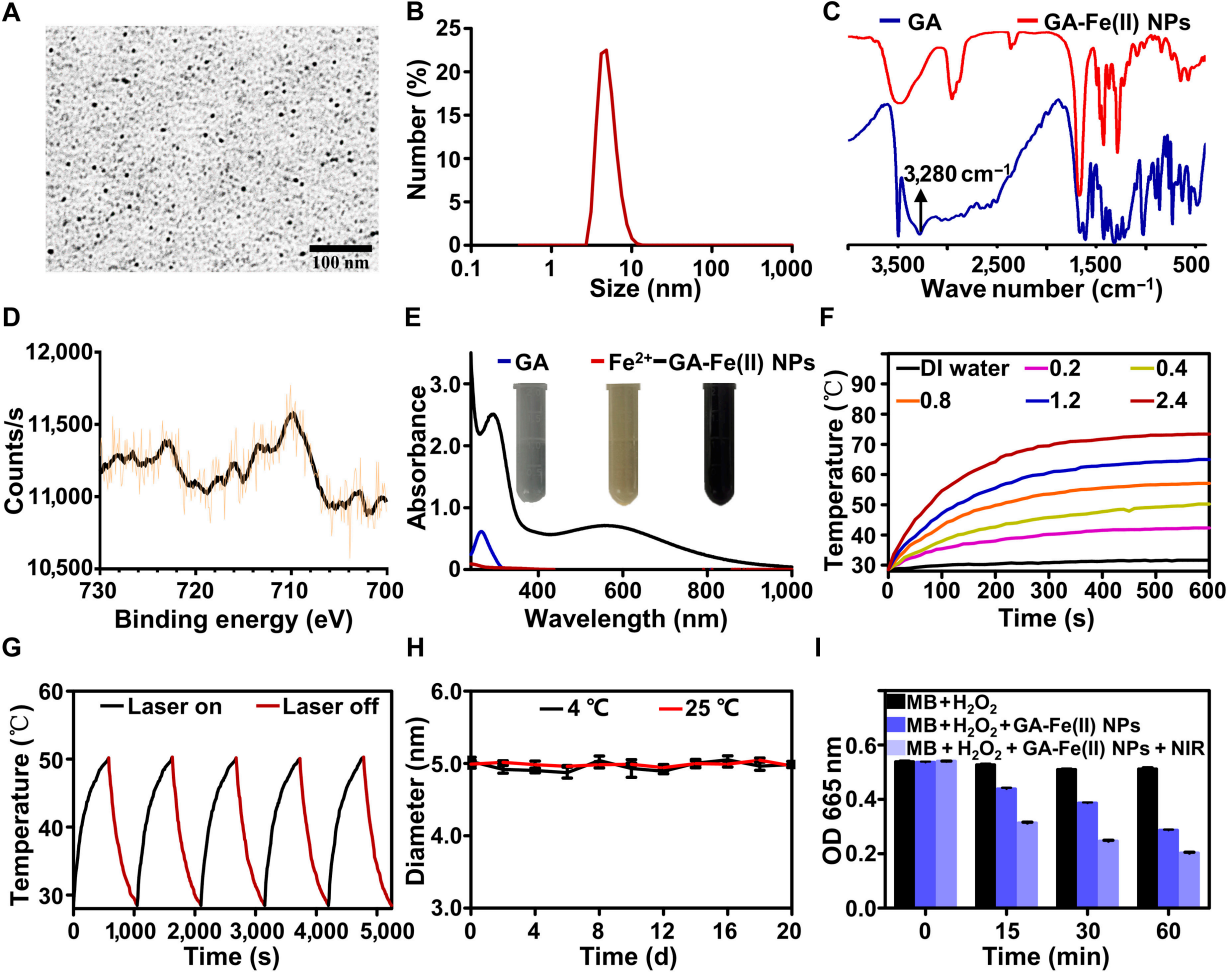
Characterization of GA-Fe(II) NPs. (A) Transmission electron microscopy (TEM) image of GA-Fe(II) NPs. (B) Diameter distribution of GA-Fe(II) NPs. (C) Fourier transform infrared (FT-IR) spectra of gallic acid (GA) and GA-Fe(II) NPs. (D) X-ray photoelectron spectroscopy (XPS) spectra of Fe 2p orbital for GA-Fe(II) NPs. (E) Ultraviolet spectra of free GA, Fe^2+^, and GA-Fe(II) NP solutions. Inset: photographs of these solutions. (F) Temperature elevation for various concentrations of GA-Fe(II) NPs with NIR laser irradiation at 1.5 W cm^−2^ for 10 min. (G) Temperature variation of GA-Fe(II) NPs (0.4 mg ml^−1^) under NIR laser irradiation at 1.5 W cm^−2^ for 5 on–off cycles. (H) Dynamic diameter change of GA-Fe(II) NPs at different temperatures after storage for a period of time. (I) Time-dependent methylene blue (MB) degradation initiated by GA-Fe(II) NP-triggered Fenton reaction with or without NIR laser exposure. DI, deionized.

Ultrasmall GA-Fe(II) NPs were successfully prepared as described in previous research. As shown in Fig. 2A, the morphology of GA-Fe(II) NPs obtained by TEM demonstrated a regular spherical structure with a homogeneous size, and the diameter was 5 nm as measured by dynamic light scattering (Fig. 2B). The doughty coordination behavior between the polyphenol groups on GA and Fe^2+^ was confirmed by a Fourier transform infrared spectrometer, showing the disappearance of the stretching band of the phenolic hydroxyl group at 3,280 cm^−1^ (Fig. 2C). Moreover, x-ray photoelectron spectroscopy analysis confirmed the presence of Fe^2+^ in GA-Fe(II) NPs, indicated by strong binding energy peaks at 711 and 724 eV, corresponding to Fe 2p_3/2_ and Fe 2p_1/2_, respectively (Fig. 2D). The proportion of Fe^2+^ in GA-Fe(II) NPs was 6% as analyzed by thermogravimetric analysis (Fig. S1).

Furthermore, the photothermal capabilities of GA-Fe(II) NPs were examined to assess their effectiveness as a photothermal agent. The ultraviolet spectrum of GA-Fe(II) NPs displayed wide absorption from 400 to 900 nm, with a peak centered at 556 nm (Fig. 2E). This finding revealed that the as-prepared GA-Fe(II) NPs, which absorb in the NIR region, can be activated by NIR lasers to capacitate PTT. Upon being subjected to an 808-nm NIR laser at 1.5 W cm^−2^, the temperature of the GA-Fe(II) NP solution increased instantly, while the temperature was almost unchanged for DI water (Fig. 2F). Meanwhile, the GA-Fe(II) NP solution exhibited concentration-dependent photothermal conversion, and the final temperature of 0.4 mg ml^−1^ GA-Fe(II) NPs reached 50.2 °C in 10 min, which was adequate for the disintegration of TSLs. Furthermore, photothermal stability was evaluated as a critical parameter for the practical application of photothermal agents. There was almost no change in the photothermal performance of GA-Fe(II) NPs after thermal-cold cycling treatment under NIR laser irradiation at 1.5 W cm^−2^, demonstrating excellent photothermal stability (Fig. 2G). Consistently, the ultraviolet spectrum of GA-Fe(II) NPs remained to have a wide absorption from 400 to 900 nm (Fig. S2), suggesting that GA-Fe(II) NPs were highly stable across multiple photothermal treatments. Moreover, the obtained GA-Fe(II) NPs exhibited long-term colloidal stability; the diameter of GA-Fe(II) NPs remained unchanged for 20 d at 4 and 25 °C, respectively (Fig. 2H).

The capacity of GA-Fe(II) NPs to produce •OH was evaluated by testing the degradation of MB as caused by •OH [35,36]. GA-Fe(II) NPs were incubated with 1 mM H_2_O_2_. As shown in Fig. 2I, the absorbance value of MB strikingly depressed in a time-dependent manner, demonstrating the degradation of MB. In contrast, MB still retained the initial absorbance in bare H_2_O_2_ alone. It is of note that the degradation of MB significantly increased after NIR irradiation, revealed by the lower absorption of MB compared to that without NIR. These results indicate that the photothermal property of GA-Fe(II) NPs led to enhancement of •OH generation, suggesting the possibility for PTT and CDT synergistic treatment.

### Characterization of the GA-Fe(II) NPs/SG@TSL antibacterial platform

**Fig. 3.**
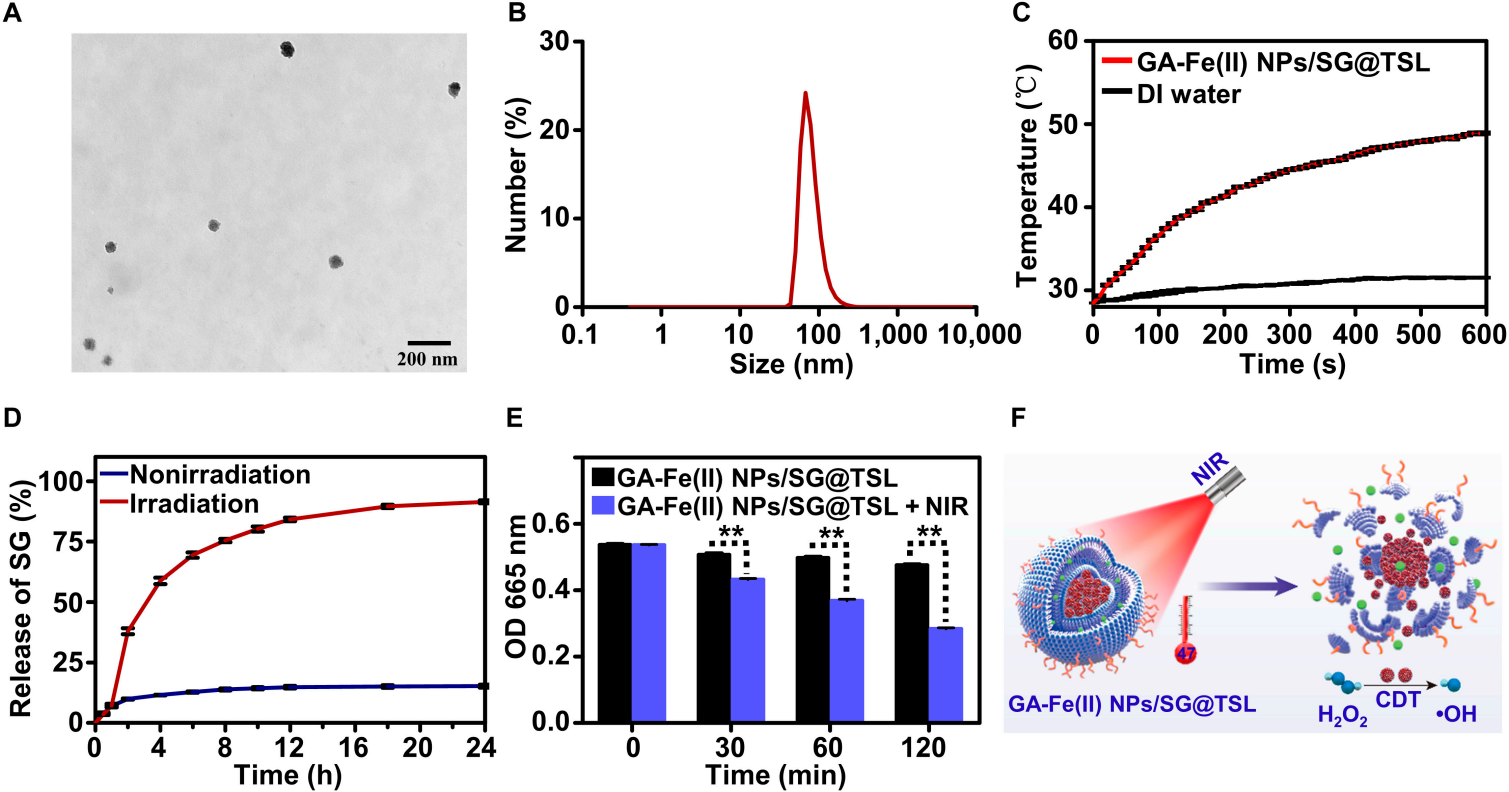
Characterization of GA-Fe(II) NPs/SG@TSL. (A) TEM image of GA-Fe(II) NPs/SG@TSL. (B) Diameter distribution of GA-Fe(II) NPs/SG@TSL measured by dynamic light scattering. (C) Temperature elevation for GA-Fe(II) NPs/SG@TSL (GA-Fe(II) NP equivalent of 0.4 mg ml^−1^) under NIR laser irradiation at 1.5 W cm^−2^ for 10 min. (D) SG release profiles from GA-Fe(II) NPs/SG@TSL, with or without NIR laser irradiation at 1.5 W cm^−2^. (E) Time-dependent MB degradation initiated by GA-Fe(II) NPs/SG@TSL-triggered Fenton reaction with or without NIR laser exposure. **P < 0.01. (F) A scheme showing that NIR-triggered GA-Fe(II) NPs/SG@TSL induced hyperthermia and subsequent rapid SG release, as well as CDT.

SG is a promising antimicrobial alkaloid, and the MIC against MRSA was 16 μg ml^−1^. It is exciting that SG could reduce the chance of resistance development compared with daptomycin and vancomycin (Fig. S3). In this study, the thin film hydration technique was used for GA-Fe(II) NPs/SG@TSL preparation owing to the possibility of its large-scale production. The TEM image revealed that the obtained GA-Fe(II) NPs/SG@TSL exhibited a spheroid morphology, and multiple smaller dark dots was observed in the interior, confirming the successful loading of GA-Fe(II) NPs (Fig. 3A). The hydrodynamic particle size of GA-Fe(II) NPs/SG@TSL was 80.2 nm with a narrow size distribution (polydispersity index = 0.181) measured by dynamic light scattering (Fig. 3B). The SG content in GA-Fe(II) NPs/SG@TSL was determined to be 67.5% ± 0.18% according to the performed ultraviolet spectrophotometry (Fig. S4 and Table S1).

The effective encapsulation of GA-Fe(II) NPs could enable GA-Fe(II) NPs/SG@TSL to be competent for photothermal performance. As we anticipated, the temperature of the GA-Fe(II) NPs/SG@TSL solution (GA-Fe(II) NP equivalent of 0.4 mg ml^−1^) increased instantly upon subjection to NIR exposure at 1.5 W cm^−2^, while negligible changes were observed in DI water, revealing that GA-Fe(II) NPs/SG@TSL could efficiently convert NIR irradiation into heat (Fig. 3C). The final temperature rose to 49 °C within 10 min, which was adequate to promote the phase transition of TSLs to release SG. Therefore, the NIR-triggered release of SG from GA-Fe(II) NPs/SG@TSL was monitored. As shown in Fig. 3D, GA-Fe(II) NPs/SG@TSL displayed a slow release of SG in the absence of NIR irradiation and the cumulative release reached only about 15% within 24 h, illustrating the integrity of the GA-Fe(II) NPs/SG@TSL system. In contrast, a rapid SG release was noted after introducing NIR irradiation on the GA-Fe(II) NPs/SG@TSL solution for 10 min at the 1-h mark, with approximately 58.8% released within 4 h. These behaviors indicated that the photothermal performance induced by GA-Fe(II) NPs/SG@TSL under NIR introduction could effectively initiate the rapid release of SG. Moreover, the GA-Fe(II) NPs/SG@TSL solution under NIR irradiation was finally stable at 47 °C, minimizing harm to adjacent healthy tissues and ensuring PTT safety [36,37]. The stability study demonstrated that GA-Fe(II) NPs/SG@TSL could well maintain its dynamic diameter and morphology in phosphate-buffered saline (pH = 7.4) after incubation for 7 d (Fig. S5A and B), indicating the stability of GA-Fe(II) NPs/SG@TSL in the physiological environment.

Furthermore, the application potential of GA-Fe(II) NPs/SG@TSL in CDT was investigated. As shown in Fig. 3E, the absorption value of MB had been roughly static in the absence of NIR irradiation. In contrast, GA-Fe(II) NPs/SG@TSL could trigger the gradual degradation of MB in the presence of H_2_O_2_ upon NIR exposure, which was similar to that of GA-Fe(II) NPs as shown in Fig. 2I. Given the above results, GA-Fe(II) NPs/SG@TSL could enable PTT, SG release, and CDT simultaneously upon exposure to NIR irradiation (Fig. 3F), which has potential for combating MDR pathogens.

### Internalization of the GA-Fe(II) NPs/SG@TSL antibacterial platform by MRSA

To track the internalization of GA-Fe(II) NPs/SG@TSL into MRSA, CM 6 was utilized as a model fluorescent drug to construct nanoparticles with TSL and GA-Fe(II) NPs. MRSA specimens incubated with GA-Fe(II) NPs/CM 6@TSL for 3 h showed a clear CM 6 fluorescence signal at all tested concentrations. Especially, MRSA treated with GA-Fe(II) NPs/CM 6@TSL (CM 6 equivalent of 0.2 μg ml^−1^) had a distinctly stronger fluorescence signal than those treated with GA-Fe(II) NPs/CM 6@TSL (CM 6 equivalent of 0.05 μg ml^−1^), suggesting that bacterial uptake of GA-Fe(II) NPs/CM 6@TSL occurred in a concentration-dependent manner (Fig. S6A). To learn more about the effect of time on bacterial uptake, bacterial accumulation of GA-Fe(II) NPs/CM 6@TSL was also quantitatively assessed using flow cytometry. As shown in Fig. S6B, MRSA treated with GA-Fe(II) NPs/CM 6@TSL displayed a significant fluorescence intensity after incubation at multiple timepoints. The counting level of GA-Fe(II) NPs/CM 6@TSL in MRSA increased as incubation time increased. These results demonstrate that the GA-Fe(II) NPs/SG@TSL antibacterial platform was able to enter MRSA to work.

### Antibacterial activities of GA-Fe(II) NPs/SG@TSL in vitro

**Fig. 4.**
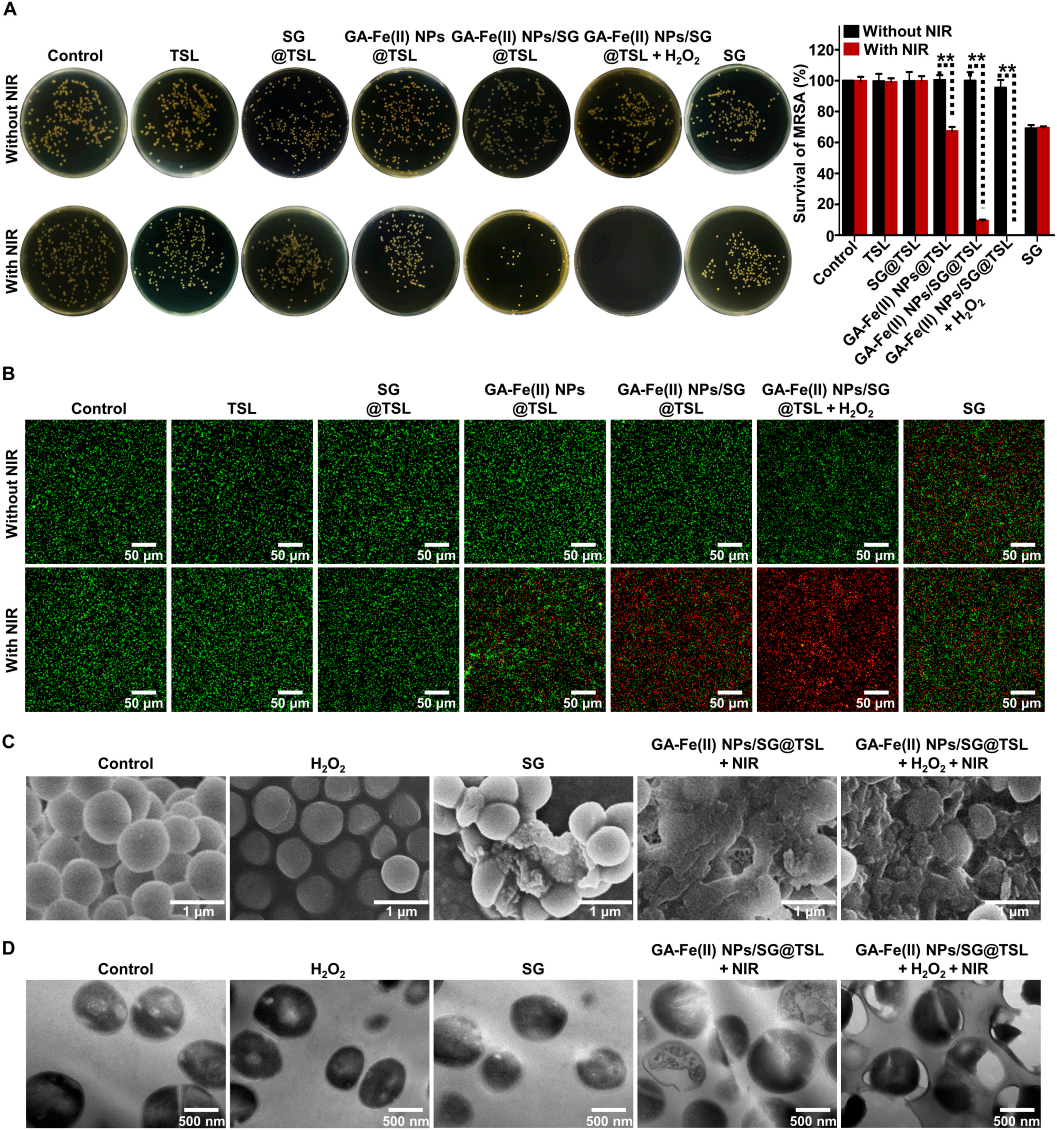
Antibacterial activities of GA-Fe(II) NPs/SG@TSL against MRSA in vitro. (A) Photographs of the colonies on trypticase soy agar (TSA) plates after different treatments and their survival rate analysis based on colony counts. **P < 0.01. (B) Inverted fluorescence microscopy photographs of live or dead MRSA after the indicated treatments. (C) Scanning electron microscopy (SEM) images and (D) TEM images of MRSA after treatment with H_2_O_2_, SG, GA-Fe(II) NPs/SG@TSL + NIR, or GA-Fe(II) NPs/SG@TSL + H_2_O_2_ + NIR.

Based on the NIR-triggered hyperthermia and subsequent rapid SG release, as well as the CDT induced by GA-Fe(II) NP catalysis, the antibacterial activity of GA-Fe(II) NPs/SG@TSL against MRSA was investigated in vitro. As shown in Fig. 4A, the antibacterial performance was quantitatively assessed using plate counting experimentation. For the administration groups without NIR irradiation, only the SG-treated group showed an obvious bactericidal effect, which indicated that NIR irradiation was necessary to potentiate the antibacterial effect of the liposomes. The survival rate of MRSA in the SG group decreased by 31%. Although promising, it did not achieve a satisfactory bactericidal effect. When NIR irradiation was introduced, an obvious reduction in the number of colonies was noted for both GA-Fe(II) NPs@TSL and GA-Fe(II) NPs/SG@TSL treatments, and the survival rates of MRSA were decreased by 32.6% and 90.6%, respectively. These results present that the photothermal effect alone exerted bactericidal activity, and the synergetic antibacterial effect was achieved by combination with SG. It is of note that the bacteria were killed upon the introduction of H_2_O_2_, attributed to the effective generation of •OH from H_2_O_2_ as demonstrated in Fig. 3E. The GA-Fe(II) NPs/SG@TSL + H_2_O_2_ + NIR group exhibited an antibacterial rate nearing 100%, with almost no visible bacterial colonies. These findings indicated that NIR-activated GA-Fe(II) NPs/SG@TSL could strongly suppress bacterial growth through the integration of hyperthermia, released SG, and boosted CDT.

The antibacterial effect of GA-Fe(II) NPs/SG@TSL was further visualized using live/dead staining assay. All bacteria with intact surfaces exhibited green fluorescence when stained with SYTO 9, whereas those bacteria with damaged walls showed red fluorescence upon staining with PI. As shown in Fig. 4B, the treated MRSA with saline, TSLs, SG@TSL, GA-Fe(II) NPs@TSL, GA-Fe(II) NPs/SG@TSL, GA-Fe(II) NPs/SG@TSL + H_2_O_2_, NIR, TSL + NIR, and SG@TSL + NIR showed strong green fluorescence, indicating the high activities of bacteria. Decreased green fluorescence and increased red fluorescence were observed in the GA-Fe(II) NPs@TSL + NIR and SG groups, indicating the partial doom of bacteria with these treatments. In contrast, MRSA treated with GA-Fe(II) NPs/SG@TSL + H_2_O_2_ + NIR exhibited the strongest red fluorescence, demonstrating complete disruption and defective cell membranes of bacteria.

The results were further demonstrated by SEM. SEM images showed that MRSA treated with GA-Fe(II) NPs/SG@TSL + NIR and SG exhibited concave holes and incurred ruptures on the surface (Fig. 4C). As for MRSA treated with GA-Fe(II) NPs/SG@TSL + H_2_O_2_ + NIR, almost visible MRSA cells underwent amorphous and fragmentized. In contrast, the MRSA cells revealed intact morphology and smooth cell surfaces in other treatment groups. A similar phenomenon was visualized in the TEM images (Fig. 4D). The GA-Fe(II) NPs/SG@TSL + H_2_O_2_ + NIR treatment resulted in significant membrane damage, evident by cell membrane fragmentation and irregularly shaped holes of MRSA. These findings suggested that the destruction of membrane was a key factor in bacterial death due to the synergistic antibacterial behaviors of GA-Fe(II) NPs/SG@TSL under NIR irradiation.

### Antibiofilm effect of GA-Fe(II) NPs/SG@TSL in vitro

**Fig. 5.**
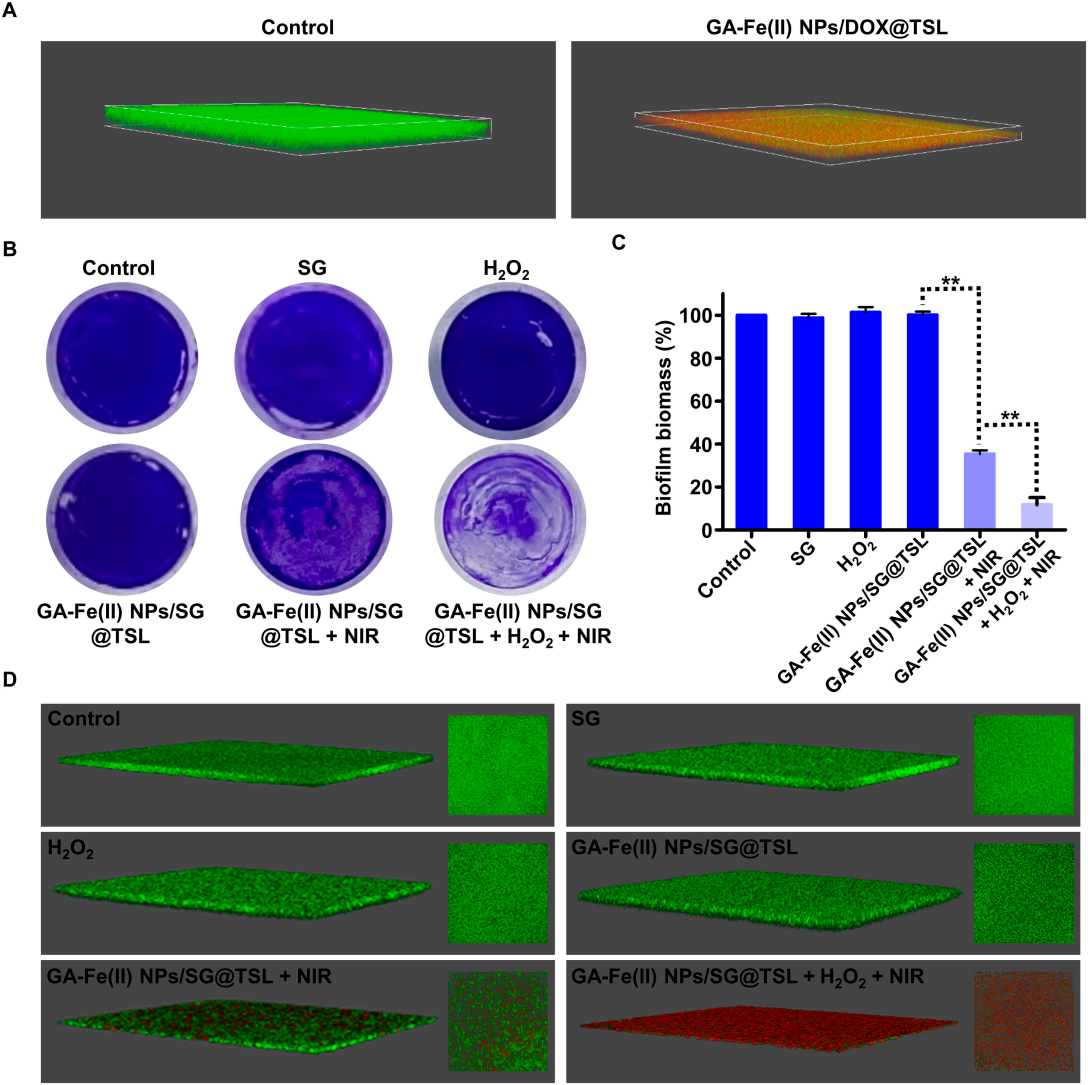
Antibiofilm effect of GA-Fe(II) NPs/SG@TSL in vitro. (A) Confocal laser scanning microscopy (CLSM) images of the penetration process of GA-Fe(II) NPs/DOX@TSL through established MRSA biofilms. MRSA biofilms labeled with SYTO 9 (green fluorescence) and TSLs loaded with the hydrophobic doxorubicin (DOX; red fluorescence). (B) Crystal violet staining and (C) biofilm biomass of MRSA biofilms following specified treatments. (D) Three-dimensional (3D) and plane CLSM images of MRSA biofilms after different treatments. MRSA biofilms and dead bacteria were tracked by SYTO 9 and propidium iodide (PI), respectively.

In MRSA infection, biofilm is a lethal part that is difficult to eliminate due to the protection of extracellular polymeric substances. The compact extracellular polymeric substances could act as barrier impeding the penetration of the antimicrobial into the biofilm, resulting in failure of bactericide treatment. Thus, the biofilm penetration ability of GA-Fe(II) NPs/SG@TSL was primarily studied using CLSM. MRSA was stained with SYTO 9 (green fluorescence), and the distribution of GA-Fe(II) NPs/SG@TSL into MRSA biofilms was tracked by loading the TSLs with the hydrophobic DOX (10 μg ml^−1^, red fluorescence). As shown in Fig. 5A, the DOX in TSLs penetrated the full layers of MRSA biofilms, as presented by a colocalization of red with green fluorescence signals, suggesting successful distribution of antibacterial agents within the biofilms.

Considering the inspiring results, we speculated that GA-Fe(II) NPs/SG@TSL possessed the ability to effectively remove MRSA biofilm. Therefore, visual and quantitative biofilm removal was studied using crystal violet staining by assessment of the relative biofilm biomass upon different treatments. As shown in Fig. 5B and C, SG displayed negligible effect on MRSA biofilm elimination due to the limited permeation. Meanwhile, the relative biomass removal rate was 64.7% for treatment with GA-Fe(II) NPs/SG@TSL + NIR. The largest extent of biofilm elimination (88.2%) was detected after GA-Fe(II) NPs/SG@TSL + H_2_O_2_ + NIR treatment. Then, live/dead double-staining assay was employed to verify biofilm eradication and subsequent bactericidal ability of GA-Fe(II) NPs/SG@TSL. Generally, MRSA biofilms and dead bacteria were tracked by SYTO 9 (green fluorescence) and PI (red fluorescence), respectively. As shown in Fig. 5D, GA-Fe(II) NPs/SG@TSL + H_2_O_2_ + NIR treatment significantly decreased the viability of bacteria in the mature biofilm. A significant presence of red fluorescence was detected on the surface as well as the depth of biofilm, indicating the outstanding antibiofilm effect and bactericidal ability. In summary, NIR-triggered GA-Fe(II) NPs/SG@TSL possessed elimination and killing ability toward mature biofilms through combining with multiple antibacterial behaviors.

### In vivo treatment of skin infections

**Fig. 6.**
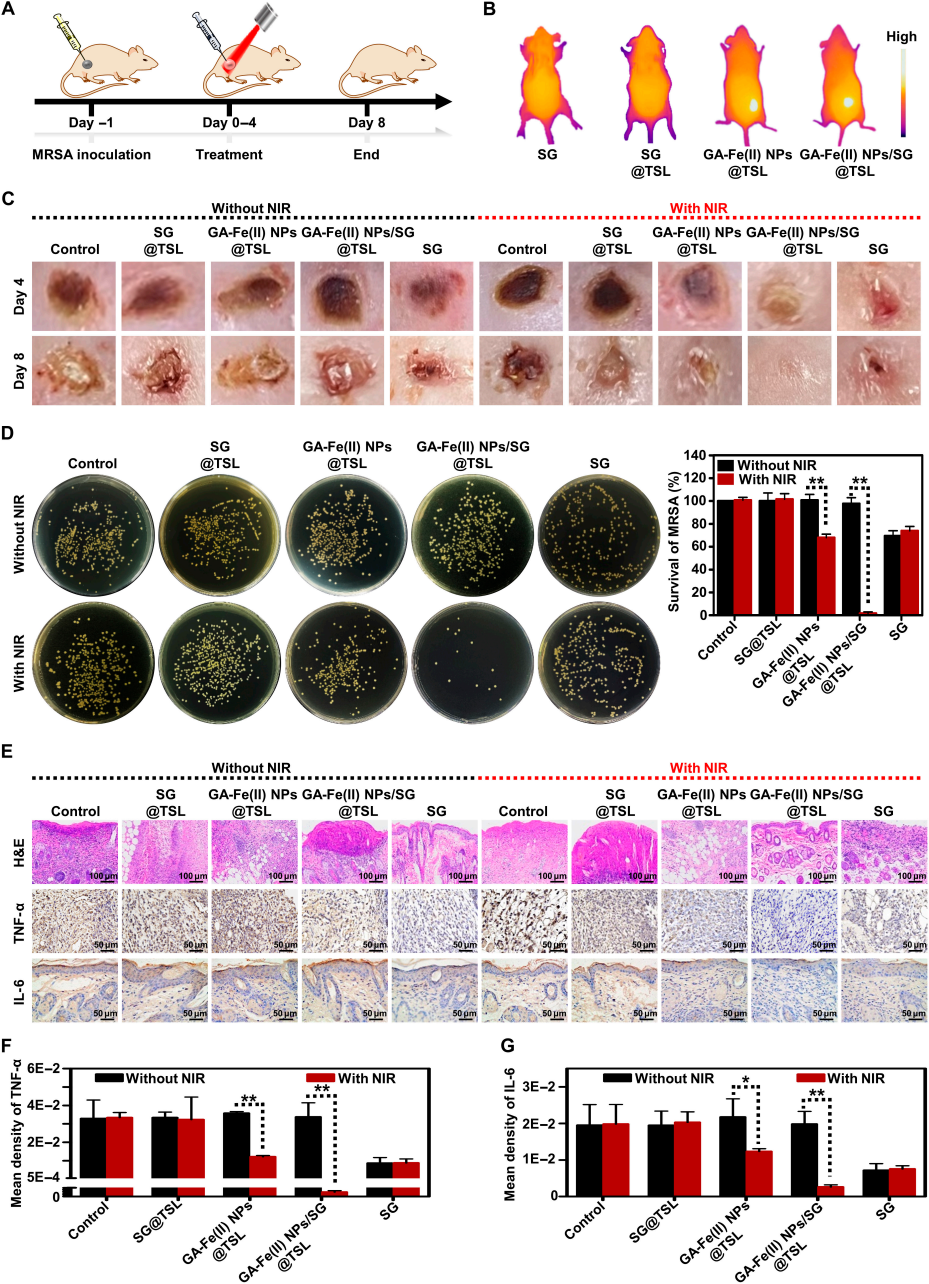
Antibacterial activities of GA-Fe(II) NPs/SG@TSL in vivo. (A) Schematic diagram of the subcutaneous abscess mouse model building and therapy process. (B) Thermal images of infected skin regions in mice treated with SG, SG@TSL, GA-Fe(II) NPs@TSL, or GA-Fe(II) NPs/SG@TSL following NIR laser exposure. (C) Images of the infected skin in mice during treatment with various formulations for 8 d. (D) Photographs of MRSA colonies from infected mouse skin under various treatments and corresponding statistical analysis of bacterial viability. **P < 0.01. (E) Histological staining and immunohistochemical staining for tumor necrosis factor alpha (TNF-α) and interleukin-6 (IL-6) of infected mouse skin after the completion of various treatments. (F and G) Quantitative analysis of immunohistochemical mean density for TNF-α and IL-6 of infected mouse skin under various treatments. *P < 0.05, or **P < 0.01. H&E, hematoxylin and eosin.

Building on the potent bacteria-killing action of NIR-triggered GA-Fe(II) NPs/SG@TSL in vitro, its antibacterial performance was further assessed using a mouse model with subcutaneous MRSA infection (Fig. 6A). The temperature of GA-Fe(II) NPs/SG@TSL at the infection region in mice rose to 47 °C following NIR irradiation (Fig. 6B and Fig. S7). The mild temperature was adequate to promote thermal therapy and release the burden while reducing tissue damage resulting from hyperpyrexia. As expected, the mice in the GA-Fe(II) NPs/SG@TSL + NIR group reached nearly complete recovery in the infected areas by the eighth day (Fig. 6C). Although a reduction in lesion size was observed in the mice with GA-Fe(II) NPs@TSL + NIR and SG treatment, the lesion was still obvious on the skin. Furthermore, the infected skin was isolated, homogenized, and spread on TSA plates to quantify the number of MRSA. No significant difference in MRSA number was found between the control group and the GA-Fe(II) NPs/SG@TSL group due to the restricted PTT and release of SG without NIR irradiation. Following treatment with SG and GA-Fe(II) NPs@TSL + NIR, the survival rates of MRSA decreased to 73% and 67.9%, respectively (Fig. 6D). Few bacteria were observed on the plate, and the survival rate of MRSA fell to 1.7% with GA-Fe(II) NPs/SG@TSL treatment after NIR introduction, demonstrating the synergistic antibacterial action of PTT, SG, and CDT.

Next, the recovery levels of infected skin were objectively assessed via histological analysis (Fig. 6E). For the control group, bad destruction of dermis structures and skin lesions were revealed from the H&E images. Moreover, no obvious recovery of skin tissues was observed in the SG@TSL, GA-Fe(II) NPs@TSL, GA-Fe(II) NPs/SG@TSL, NIR, and SG@TSL + NIR groups. The results were similar to those in Fig. 6C. In contrast, the skin tissues from GA-Fe(II) NPs/SG@TSL + NIR treatment exhibited more intact histological features, followed by SG and GA-Fe(II) NPs@TSL + NIR treatment. Aside from effective MRSA killing, relieving excessive inflammation is an essential process for bacterial infection therapy. Thus, the effect of NIR-triggered GA-Fe(II) NPs/SG@TSL treatment on local inflammation was studied by immunohistochemical staining (Fig. 6E to G). Although SG showed lower bactericidal activity than GA-Fe(II) NPs@TSL + NIR treatment in vivo, a stronger anti-inflammatory effect was observed. The proinflammatory factor levels of infected tissues, including TNF-α and IL-6 levels, in the SG group were reduced by more than 70% and 60%, respectively. In fact, the anti-inflammatory behavior of SG has been demonstrated previously [38]. The multiple pharmacological activities further highlight the potential of SG in MRSA infection treatment. Interestingly, benefiting from the multiple combination action of PTT, SG, and CDT, GA-Fe(II) NPs/SG@TSL + NIR treatment resulted in remarkably reducing the TNF-α and IL-6 levels by 99% and 87% in mice with subcutaneous abscesses, respectively. Collectively, the findings clearly demonstrate that NIR-triggered GA-Fe(II) NPs/SG@TSL can exert a double sword of antibacterial and anti-inflammatory effects to efficiently beat MRSA infection. Encouraged by this, the antibiotic substitution potential of NIR-triggered GA-Fe(II) NPs/SG@TSL was further studied by comparing their therapeutic effect with vancomycin. The GA-Fe(II) NPs/SG@TSL + NIR group had therapeutic efficacy almost equivalently high with that of the vancomycin group, as demonstrated by the analogous abscess recovery and obvious reduction of MRSA colony numbers (Fig. S8A to C). This result implied that the antibacterial effect of NIR-triggered GA-Fe(II) NPs/SG@TSL presented potential application prospects.

### In vivo biosafety of GA-Fe(II) NPs/SG@TSL

The cytocompatibility of TSLs was first evaluated by testing their toxicity on L929 cells using the CCK-8 assay. The viability of L929 cells was still above 95% even when the TSL concentration was up to 2 mg ml^−1^ (Fig. S9A). Moreover, a hemolysis assay was further performed. When the TSL concentration was 2 mg ml^−1^, the supernatants of the TSL group was transparent and the hemolysis ratio could still be maintained at about 1.5% (Fig. S9B). These results demonstrated that TSL had good cytocompatibility and exerted negligible damage to RBCs.

To further explore the potential clinical application of GA-Fe(II) NPs/SG@TSL, its biosafety was evaluated in vivo. The administration procedure and treatment period of each group followed the treatment process in vivo. Compared with healthy mice, there were no differences in the white blood cell, granulocyte, hematocrit, platelet, RBC, lymph, mean corpuscular volume, mean corpuscular hemoglobin, mean corpuscular hemoglobin concentration, and hemoglobin levels of the mice treated with GA-Fe(II) NPs/SG@TSL + NIR, indicating the lack of any clear impact of blood parameters (Fig. S10A). Meanwhile, blood biochemical analysis showed that no appreciable changes in primary biomarkers of liver function (alanine aminotransferase and aspartate aminotransferase) or kidney function (blood urea nitrogen and creatinine) were observed (Fig. S10B), indicating that GA-Fe(II) NPs/SG@TSL + NIR treatment exerts negligible liver toxicity and nephrotoxicity. Furthermore, H&E images of GA-Fe(II) NPs/SG@TSL + NIR treatment displayed no appreciable damages or histological changes in major organs compared with those of normal mice (Fig. S10C). Thus, it could be concluded that NIR-triggered GA-Fe(II) NPs/SG@TSL possesses superior biosafety.

### The antibacterial mechanism of GA-Fe(II) NPs/SG@TSL

**Fig. 7.**
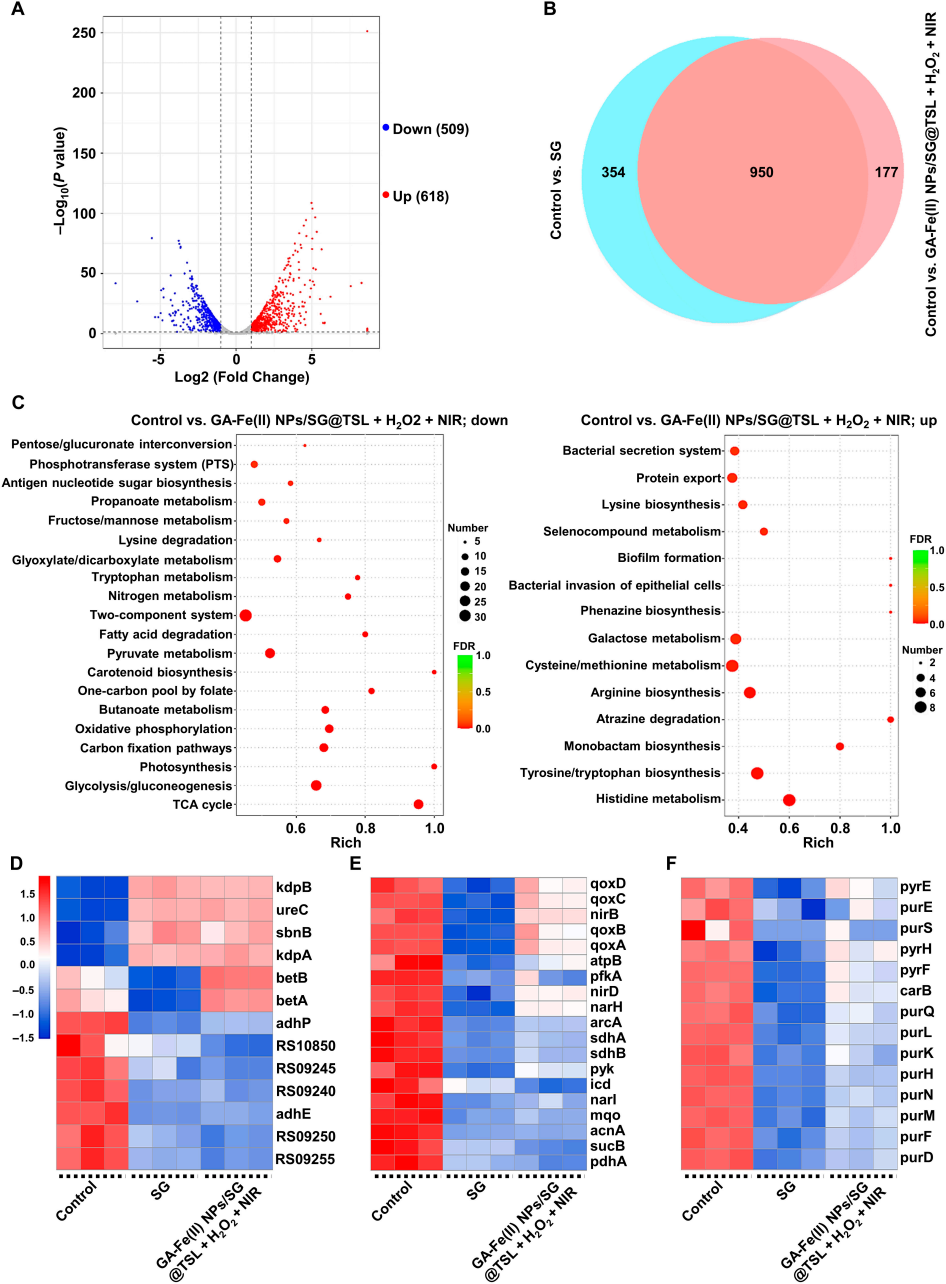
Exploration of multiple antibacterial mechanisms of GA-Fe(II) NPs/SG@TSL. (A) Volcano plots showing the identified up-regulated and down-regulated genes by GA-Fe(II) NPs/SG@TSL + H_2_O_2_ + NIR treatment. (B) Venn diagram showing the number of differentially expressed genes (DEGs) for control versus SG and control versus GA-Fe(II) NPs/SG@TSL + H_2_O_2_ + NIR treatment. (C) Kyoto Encyclopedia of Genes and Genomes (KEGG) enrichment for the identified DEGs of control versus GA-Fe(II) NPs/SG@TSL + H_2_O_2_ + NIR treatment. (D) Heatmap of DEGs in cell membrane damage after SG or GA-Fe(II) NPs/SG@TSL + H_2_O_2_ + NIR treatment. (E) Heatmap of DEGs in the energy metabolism pathway after SG or GA-Fe(II) NPs/SG@TSL + H_2_O_2_ + NIR treatment. (F) Heatmap of DEGs in nucleic acid synthesis after SG or GA-Fe(II) NPs/SG@TSL + H_2_O_2_ + NIR treatment. TCA, tricarboxylic acid; FDR, false discovery rate.

To acquire a deep cognizance of the antibacterial mechanisms of NIR-triggered GA-Fe(II) NPs/SG@TSL, transcription analysis of MRSA was performed after exposure to SG or GA-Fe(II) NPs/SG@TSL + H_2_O_2_ + NIR in vitro. About 1,304 DEGs were recorded between control and SG groups (Fig. S11A). A total of 1,127 DEGs with significant expression patterns before and after GA-Fe(II) NPs/SG@TSL + H_2_O_2_ + NIR treatment were identified (Fig. 7A). Among them, 950 DEGs were found to be shared with SG treatment, and 177 DEGs were unique in GA-Fe(II) NPs/SG@TSL + H_2_O_2_ + NIR treatment, which demonstrated that the antibacterial mechanisms of NIR-triggered GA-Fe(II) NPs/SG@TSL were contributed by SG and other additional actions (Fig. 7B). KEGG enrichment analysis presented that these shared DEGs were enriched in cell membrane damage. Generally, cell membrane damage of bacteria often causes osmotic imbalance, which triggers a response to the accumulation of alternative osmolytes. It was observed that several key amino acids involved in osmotic adjustment, including histidine and methionine, were up-regulated in their metabolic pathways (Fig. 7C and Fig. S11B). In addition, proline is considered to play a crucial role in osmotic adjustment. The overexpression of the *sbnB* gene, which is analogous to ornithine cyclodeaminase, enhanced proline synthesis (Fig. 7D) [39]. Another important finding from membrane-related DEGs was up-regulation of genes like the ones encoding potassium-transporting ATPase (*kdpA* and *kdpB*), which contributed to osmotolerance, indicating the supervision of osmotic pressure in MRSA [40]. Furthermore, exposure to SG or GA-Fe(II) NPs/SG@TSL + H_2_O_2_ + NIR altered membrane components and function pathways in MRSA, particularly affecting the fatty acid degradation pathway. This result indicated that MRSA mitigated their membrane damage by inhibiting fatty acid degradation (Fig. 7C and Fig. S11B) [41,42]. In addition, a reduction in intracellular pH serves as an indicator of membrane damage. Consequently, MRSA underwent protective adjustments to maintain pH homeostasis when subjected to SG or GA-Fe(II) NPs/SG@TSL + H_2_O_2_ + NIR treatments. On the one hand, the genes associated with acid-generating pathways, including *adhE* and *adhP* in the fermentative pathway, were down-regulated. On the other hand, a gene of *ureC* related to urease was detected to be overexpressed, which could contribute to favoring intracellular pH homeostasis (Fig. 7D) [39]. These results indicated that SG and GA-Fe(II) NPs/SG@TSL + H_2_O_2_ + NIR treatment exerted an antibacterial effect by inducing cell membrane damage. Interestingly, the same antibacterial mechanism of CDT has already been well established in previous literature. As demonstrated, CDT is based on the Fenton reaction, which decomposes H_2_O_2_ to generate toxic •OH to destroy the bacterial membrane, leading to leakage of intracellular contents and further bacterial cell death [43]. Taken together, SG release and •OH generation of GA-Fe(II) NPs/SG@TSL could be simultaneously triggered upon NIR irradiation. Importantly, both SG and toxic •OH could destroy the bacterial membrane, thus achieving a synergistic antibacterial effect. Accordingly, GA-Fe(II) NPs/SG@TSL + H_2_O_2_ + NIR treatment caused the worst damage to MRSA membranes and leakage of contents due to the introduction of CDT (Fig. 4D).

On closer inspection, it was found that the energy metabolism and nucleic acid synthesis in MRSA were highly enriched with the shared DEGs between SG and GA-Fe(II) NPs/SG@TSL + H_2_O_2_ + NIR treatments. The DEG heatmap demonstrated that most genes were significantly down-regulated in these pathways (Fig. 7E and F). Specifically, the major energy metabolism pathways, including glycolysis, tricarboxylic acid cycle, pyruvate metabolism, oxidative phosphorylation, and nitrogen metabolism, were obviously repressed (Fig. 7C and Fig. S11B) [40,41,44,45]. In regard to nucleic acid synthesis, we observed clear inhibition of the *pur* and *pyr* operons, which are crucial to RNA and DNA biosynthesis [46]. Synchronously, *carB*, a key enzyme of the nucleotide rescue pathway, was also repressed, resulting in a blockage of the salvage synthesis of pyrimidine nucleotides (Fig. 7F) [47]. The signs from the above indicated that NIR-triggered GA-Fe(II) NPs/SG@TSL induced energy metabolism disorder and inhibited nucleic acid synthesis in MRSA.

Signally, several pathways were regulated only in GA-Fe(II) NPs/SG@TSL + H_2_O_2_ + NIR treatment. It is well established that lysine is used for the tetrapeptide side chains of the *S. aureus* wall [48]. The compensation behaviors that synchronously occurred in the enhanced lysine biosynthesis pathway and repressed lysine degradation pathway explained the inhibitory effect of GA-Fe(II) NPs/SG@TSL + H_2_O_2_ + NIR treatment on cell wall synthesis (Fig. 7C). Bacteria take up and metabolize carbohydrates for producing energy or partaking in bacterial component biosynthesis pathways. The phosphotransferase system (PTS) has been known as the main uptake mechanism of carbohydrates by bacteria [49]. This study showed that the PTS in MRSA was repressed under GA-Fe(II) NPs/SG@TSL + H_2_O_2_ + NIR stress (Fig. 7C), which may lead to carbohydrate metabolism disorders. One of the most direct evidence to support this view was that fructose and mannose, commonly transported across the cell membrane by the PTS, and their metabolic pathways are down-regulated (Fig. 7C). Furthermore, the down-regulated pathways that are intimately correlated with carbohydrate metabolism, including propanoate metabolism and glyoxylate and dicarboxylate metabolism, consolidated the view of carbohydrate metabolism disorders (Fig. 7C).

## Conclusion

In summary, a thermally responsive antibiotic-free system was constructed for effective treatment of MRSA infections. The resultant GA-Fe(II) NPs/SG@TSL system possessed good biocompatibility. Upon NIR laser irradiation, it could not only introduce local hyperthermia and •OH but also promote spatiotemporal SG release. Taking the superiorities of physical attack via PTT, phytochemical sterilization derived from SG, and chemical damage via CDT, the system effectively killed planktonic MRSA and eradicated MRSA biofilm in vitro. The system exhibited an bactericidal effect by several important pathways, including damage of the cell membrane, inducing energy metabolism disorder, inhibition of nucleic acid synthesis, etc. After administration in an MRSA-induced subcutaneous abscess, GA-Fe(II) NPs/SG@TSL exhibited superior effectiveness of synergistic disinfection at a low SG dose, thus reducing the adverse effect. This work highlights a practicable antibiotic-free strategy to treat MRSA infection, which has the potential to be developed into a universal natural product delivery platform for efficient and safe application in MDR infections.

## Ethical Approval

The experimental animal research ethics committee of Northeast Agricultural University (SRM-11) approved the animal experiment protocols, ensuring compliance with relevant ethical regulations.

## Data Availability

All data necessary to support these findings are in the paper or in Supplementary Materials.
