# Supplementary material for: Phytochemical-Loaded Thermo-responsive Liposome for Synergistic Treatment of Methicillin-Resistant Staphylococcus aureus Infection
Source: Biomater Res. 2025 Mar 13;29:0159. doi: 10.34133/bmr.0159 (PMC11906118; doi:10.34133/bmr.0159)
Supplement: Supplementary 1 — Figs. S1 to S11 Table S1 [file bmr.0159.f1.docx]

**Phytochemical-Loaded Thermo-Responsive Liposome for Synergistic Treatment of MRSA Infection**

**Sidi Zheng^1,2^, Xinshu Zou^1,2^, Yanru Wei^1,2^, Xilong Cui^1,2^, Shuang Cai^1,2^, Xiubo Li^3^, Zhiyun Zhang^1,2*^, Yanhua Li^1,2*^**

^1^College of Veterinary Medicine, Northeast Agricultural University, Harbin 150030, PR China.

^2^Heilongjiang Key Laboratory for Animal Disease Control and Pharmaceutical Development, Harbin 150030, PR China.

^3^Feed Research Institute, Chinese Academy of Agricultural Sciences, Beijing 100081, PR China.

*****Address correspondence to: [zhangzhiyun@neau.edu.cn](mailto:zhangzhiyun@neau.edu.cn) (Z. Zhang); [liyanhua@neau.edu.cn](mailto:liyanhua@neau.edu.cn) (Y. Li)

**Figure S1.** TGA curves of GA and GA-Fe(II) NPs.

**Figure S2.** Ultraviolet spectrum of GA-Fe(II) NPs (0.4 mg mL^-1^) with or without an 808 nm laser irradiation (1.5 W cm^-2^) for 10 min.

**Figure S3.** Resistance development during serial passaging in the presence of sub-MIC concentrations of antimicrobials. Daptomycin and vancomycin served as the positive control. Each point represents the average value of three independent trials.

**The methodological verification of SG**

**Linearity**

The linearity of the proposed method was evaluated within the SG concentration range. As can be seen from the **Fig. S4**, the linear dynamic range is 0.0429x+0.0472 (R^2^=0.9987), and SG exhibited good linearity in the range of 2-16 µg mL^-1^.

**Figure S4.** The standard curve of SG.

**Precision**

The inter-day precision and intra-day precision was investigated by analyzing low, medium and high concentrations of SG on three days. The SG showed good inter-day precision and intra-day precision, and the corresponding RSDs range is 1.1−1.4% and 1.0−1.8%, respectively (**Table S1**).

**Recovery**

The absorbance of the empty TSL solution containing low, medium and high concentrations of SG was detected by UV-visible spectrophotometer, and the recovery was calculated. The obtained recoveries were above 98%, and the RSDs were less than 2% at all sample, indicating the proposed method meets the experimental requirements (**Table S1**).

**Table S1.** Linearity, precision and recovery of ultraviolet spectrophotometry analysis.

|  | Linear range  (µg mL^-1^) | Coefficient of determination | Inter-day  RSD (%) | Intra-day  RSD (%) | Recovery  RSD (%) |
| --- | --- | --- | --- | --- | --- |
| SG | 2−16 | 0.9987 | 1.1−1.4 | 1.0−1.8 | *<*2 |

**Figure S5.** The stability of GA-Fe(II) NPs/SG@TSL in the PBS (pH=7.4) at 37 ℃. (A) Dynamic diameter changes within 7 days. (B) TEM image on the 7^th^ day.

**Figure S6.** Internalization ability of GA-Fe(II) NPs/CM 6@TSL by MRSA. (A) Mean fluorescence intensity of MRSA incubated with GA-Fe(II) NPs/CM 6@TSL with different CM 6 dosage for 3 h. (B) Mean fluorescence intensity of MRSA incubated with GA-Fe(II) NPs/CM 6@TSL with equivalent CM 6 dosage (0.2 μg mL^-1^) for different intervals.

**Figure S7.** Temperature elevation of skin-infected mice injected with SG, SG@TSL, GA-Fe(II) NPs@TSL or GA-Fe(II) NPs/SG@TSL, after NIR laser irradiation.

**Figure S8.** (A) Images of the infected skin during treatment with various formulations for 8 days. (B) Photographs of MRSA colonies from infected mouse skin under various treatments and (C) corresponding statistical analysis of MRSA viability.


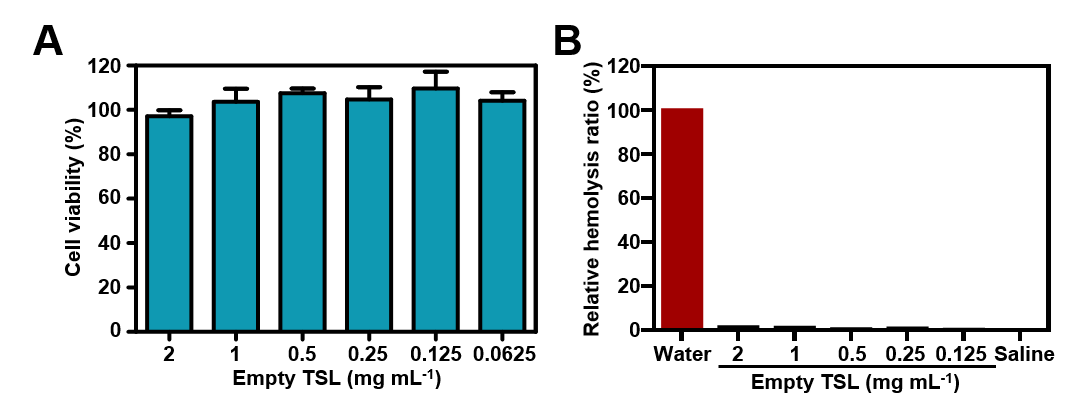


**Figure S9.** Toxicity evaluation of empty TSL. (A) Cell viability of L929 cells after incubation with various empty TSL concentrations for 24 h. (B) Relative hemolysis ratio of sheep red blood cells after incubation with water, saline, or different concentrations of empty TSL for 3 h.

**Figure S10.** *In vivo* biosafety evaluations of GA-Fe(II) NPs/SG@TSL. (A) Blood hemanalysis of WBC, Gran, HCT, PLT, RBC, lymph, MCV, MCH, MCHC and HGB in mice at completion of the treatment with saline, SG or GA-Fe(II) NPs/SG@TSL. (B) Blood biochemical analysis of ALT, AST, BUN and CRE in mice at completion of the treatment with saline, SG or GA-Fe(II) NPs/SG@TSL. (C) Histological analysis of liver, kidney and spleen from mice at the end of treatment with saline, SG or GA-Fe(II) NPs/SG@TSL.

**Figure S11.** Exploration of multiple antibacterial mechanisms of GA-Fe(II) NPs/SG@TSL. (A) Volcano plots showed the identified up-regulated and down-regulated genes by SG treatment. (B) KEGG enrichment for the identified DEGs of control versus SG treatment.
